# Current Research Approaches and Challenges in the Obesogen Field

**DOI:** 10.3389/fendo.2019.00167

**Published:** 2019-03-22

**Authors:** Raquel Chamorro-Garcia, Bruce Blumberg

**Affiliations:** ^1^Department of Developmental and Cell Biology, University of California, Irvine, Irvine, CA, United States; ^2^Department of Pharmaceutical Sciences, University of California, Irvine, Irvine, CA, United States; ^3^Department of Biomedical Engineering, University of California, Irvine, Irvine, CA, United States

**Keywords:** obesogens, obesity, MSCs, 3T3-L1, transgeneration, isoDMBs, tributyltin, metabolism

## Abstract

Obesity is a worldwide pandemic that also contributes to the increased incidence of other diseases such as type 2 diabetes. Increased obesity is generally ascribed to positive energy balance. However, recent findings suggest that exposure to endocrine-disrupting chemicals such as obesogens during critical windows of development, may play an important role in the current obesity trends. Several experimental approaches, from *in vitro* cell cultures to transgenerational *in vivo* studies, are used to better understand the mechanisms of action of obesogens, each of which contributes to answer different questions. In this review, we discuss current knowledge in the obesogen field and the existing tools developed in research laboratories using tributyltin as a model obesogen. By understanding the advantages and limitations of each of these tools, we will better focus and design experimental approaches that will help expanding the obesogen field with the objective of finding potential therapeutic targets in human populations.

In the last 40 years obesity rates have dramatically increased worldwide both in adults and in youth (1). A recent report on obesity trends in the U.S. estimated that 39.8% of adults are clinically obese (BMI > 30) (1). Importantly, obesity is a risk factor for other metabolic disorders such as type 2 diabetes whose prevalence has increased in parallel with obesity and which is predicted to affect 642 million people worldwide by 2040 (2, 3). The health costs associated with obesity were estimated at over $275 annually in the U.S alone (4). Although the major driving factor in obesity is usually considered to be a simple function of energy balance (calorie consumption higher than calorie expenditure) (5), recent reports showing the increasing trends of obesity in children under 1 year of age and in animal populations suggest that other factors may be playing important roles in obesity (6, 7). Understanding those factors, the windows of susceptibility and the mechanisms through which they to alter human metabolism and promote obesity will aid treating and preventing the increasing rates of obesity and related disorders worldwide.

Obesity is sexually dimorphic (8). Overall, females accumulate more fat than males, and it tends to be located subcutaneously, while in males, adipose tissue tends to accumulate in the visceral cavity. Subcutaneous adipose tissue is generally associated with healthier fat since it is involved in regulating thermogenesis while visceral adipose tissue is associated with increased risk of cardiovascular disease (9). Adipose tissue is now known not only for its ability to store lipids, but also for its contribution to metabolic homeostasis by secreting hormones (e.g., leptin and adiponectin) and other signaling molecules involved in the regulation of appetite and fat mobilization. This suggests that maintaining a healthy and functional adipose tissue improves the overall metabolic state of the individual (9). The sexually dimorphic content and distribution of fat is highly influenced by steroid hormones such as testosterone and estrogens (8). Therefore, alterations of the endocrine system may contribute to disturbances in the regulation of adipose tissue formation and maintenance.

Endocrine disrupting chemicals (EDCs) are exogenous chemicals that alter the natural function of hormones (10). Several human and animal studies have found associations between exposure to EDCs and increased adiposity (11–16). However, the effects of EDCs that may lead to obesity might not be linked exclusively to life style or environmental exposures during adulthood. Epidemiological studies have shown that the environment during *in utero* development may affect the prevalence of non-communicable diseases, including metabolic disorders, later in life (13–21). Notably, the nutritional state of the mother is strongly linked to body weight of the offspring at birth and later in life (22–26) suggesting that suboptimal environments during development may contribute to permanent alterations in the individual that will counteract any lifestyle actions to ameliorate weight gain, thereby increasing susceptibility to obesity (22, 27).

A large body of evidence shows that exposure to environmental pollutants during *in utero* development and early life may contribute to obesity later in life (11). Studies performed using both *in vitro* and *in vivo* models showed that a subset of endocrine disrupting chemicals, called obesogens, can have important effects on the development of adipose tissue. Obesogens are exogenous chemicals that inappropriately stimulate adipogenesis and fat storage, can disturb adipose tissue homeostasis and affect metabolic rates and/or the regulation of appetite and satiety (28). Our recent findings suggested that one consequence of these disturbances is the alteration of the metabolic setpoint of the individual, that is, the capability of the body maintain a particular weight regardless of the lifestyle changes taken to increase or reduce that weight (29). These results are supported by human studies showing an association between higher levels of perfluoroalkyl compounds in plasma and a more rapid weight regain after diet-induced weight loss plan (12). Moreover, individuals with the highest levels of perfluoroaklyl compounds has the lowest resting metabolic rate (12). Despite numerous studies demonstrating the existence and effects of obesogens, a longstanding debate about the mechanisms through which obesogens exert their obesogenic effects still persists. In this review, we discuss the current experimental approaches to study the mechanisms of function of obesogens from *in vitro* analysis to transgenerational *in vivo* studies.

## *In vitro* Approaches

The prototypical obesogen tributyltin (TBT) is known to activate two nuclear receptors critical for adipogenesis. These are the peroxisome proliferator-activated receptor gamma (PPARγ)(30–33), which is considered to be the master regulator of adipogenesis (34), and its heterodimeric partner the retinoid X receptor (RXR)(30), which we recently showed to be an important regulator of adipocyte commitment (35). Adipogenic commitment is the process through which multipotent mesenchymal stem cells (MSC) lose their multipotency and become irreversibly destined to the adipocyte lineage, in part via the expression of PPARγ2. Activation of PPARγ2 elicits terminal differentiation into white adipose tissue (36). Other already characterized obesogens such as fludioxonil, quinoxyfen, dibutyltin or triphenyltin, also activate PPARγ and/or RXR (37, 38), suggesting that these nuclear receptors are common targets for EDCs. However, nuclear receptors other than PPARγ2 and RXR have been shown to play important roles in adipogenesis, including the glucocorticoid receptor (GR) (39), estrogen receptor (ER) (40) and androgen receptor (AR) (41) and obesogens such as tolylfluanid, bisphenol A (BPA) or dichlorodiphenyltrichloroethane (DDT) have been shown to act through them (42–44). The fact that estrogen and androgen receptors are also involved in regulation of adipogenesis and fat storage, may explain, at least in part, the sexually dimorphic distribution of the fat in individuals from different genders. Therefore, assessing the capability of a candidate obesogen to induce adipogenic commitment, its ability to promote final adipocyte differentiation, and the mechanisms through which these processes occur requires the use of an array of assays that tests the various potential paths used by obesogens to promote fat storage.

One frequently used tool to screen for new obesogens is the murine pre-adipocyte cell line 3T3-L1 (45). This immortalized cell line has some beneficial aspects that include the short length of the assay and the amenability of cell lines to high throughput screening approaches. However, one key limitation of 3T3-L1 cells is that, since they are already pre-adipocytes, they are not useful for examining mechanisms, including screening for chemicals that may be involved in adipocyte commitment. It was recently suggested that the commercial source of the cell line (e.g., ATCC or Zenbio), the type of plates used, as well as the number of passages the cells have experienced play a critical role in the process of cell differentiation (46). 3T3-L1 cells from different commercial sources showed different expression of nuclear receptors such as PPARγ, RXRβ, and ERβ, and variable capability to differentiate into adipocytes using well-established adipogenic positive controls such as rosiglitazone (PPARγ activator) or TBT (46). These findings highlight some of the limitations of using 3T3-L1 cells as an adipogenic model when the goal is to hypothesize about *in vivo* outcomes after exposure to obesogens. Screening of new obesogens using this model will depend critically on poorly controlled variables such as the source and number of passages of the cells, bovine sera, inconsistencies in coatings on tissue culture plates and other culture conditions which can confound reproducibility of the results.

In contrast, assays performed with uncommitted cells such as MSCs (47), allow analyses with a broader scope. We recently described for the first time the role of RXR in adipogenic commitment using MSCs as a cell model, revealing that this phase of adipogenesis can be targeted by obesogens (35). Others have used murine cell lines such as C3H/10T1/2 which resemble MSCs in some properties (but contain a karyotype of 80 chromosomes), or BMS2 to screen for new obesogens or to dive into adipogenic mechanisms through which obesogens may act. Although both cell types are accepted adipogenic models (48, 49), they are both immortalized cell lines that do not fully recapitulate the behavior of uncommitted precursors *in vivo*. Both are also murine cells which can somewhat limit the extrapolation of results to other species. Therefore, conclusions and inferences made from the use of these cells should account for such limitations. One advantage of using MSCs is that they can be isolated as primary cells from different individuals in a variety of species allowing a more extensive analysis. The use of human MSCs can also allow researchers to assess the variability that exists in human populations, which would contribute to a deeper understanding of the role obesogens may play in the current obesity pandemic.

## *In vivo* Approaches

TBT has been shown to act as an obesogen in multiple organisms including mice (30–32, 50–52) rats (53), goldfish (54), and zebrafish (55) which supports the hypothesis that obesogens are largely not species specific. However, although there are several *in vitro* models available to study the molecular underpinnings of obesogen exposure, the mechanisms through which obesogens contribute to obesity in animal models are likely to be more complex than those described for cell cultures. Experiments performed in adult rodents exposed to TBT showed alterations of the hypothalamic-pituitary-gonadal axis (56) as well as non-alcoholic fatty liver (52) supporting the current knowledge that the regulation of metabolism requires coordination between different organs including fat tissue, liver, muscle, pancreas, or brain.

Another source of discussion in the obesogen field is related to the functionality of the adipocytes produced after exposure to obesogens (57). As mentioned above, the adipose tissue has an important role to play in maintaining an overall healthy metabolic state (58). Interestingly, white fat cells produced by TBT exposure lack some beneficial properties of normal white adipocytes since the former show reduced levels of the antidiabetic hormone adiponectin, reduced glucose uptake capability, impaired ability to develop into thermogenic beige/brite adipocytes and increased expression of pro-inflammatory and pro-fibrotic genes (57, 59, 60). These results suggest that TBT promotes the development of dysfunctional adipocytes (57, 59, 60). Understanding which obesogens produce adipocytes of normal function and which elicit the production of dysfunctional adipocytes may also contribute to a better understanding of the potential mechanistic differences between different obesogens.

## Transgenerational Approaches

It has been shown that some obesogens including TBT, DDT, and BPA, are able to induce heritable changes that are propagated through multiple generations without any further exposure (29, 61–64). The non-Mendelian transmission of these alterations suggests that epigenetic mechanisms are involved in this process. This raises yet another challenge for the obesogen field: what are the mechanisms underlying transgenerational inheritance?

The field of environmental epigenetics studies the epigenetic alterations introduced by environmental factors that contribute to phenotypic variation and/or disease (65). Studies performed in animal models showed that exposure to obesogens during development increased predisposition to obesity not only in the directly exposed F1 and F2 generations (F2 are exposed as germ cells inside the F1 fetus) but also in subsequent F3 and F4 generations (and beyond) which are not exposed (29, 61–64, 66, 67). These studies showed alterations of epigenetic factors such as DNA methylation and post-translational histone modifications in somatic tissues and/or germ cells (29, 61–64, 66–68). However, there are key knowledge gaps in this transgenerational puzzle, including the characterization of the molecular mechanisms that occur in the directly exposed stages that trigger the transgenerational phenotype, and the molecular mechanisms by which these alterations are transmitted through multiple generations.

There is extensive epigenetic reprogramming at different stages of mammalian development. Shortly after fertilization, the first erasure of epigenetic marks (DNA methylation) occurs from the zygote to the blastocyst during maternal-to-zygotic transition in a sex-specific manner (69). Between E 6.5 and E 13.5, after somatic and germ lines separate, the primordial germ cells (PGCs) undergo another round of DNA methylation erasure while they travel to the genital ridge where they will mature into sex-specific germ cells (70). Considering that from the moment of fertilization until birth there is an extensive epigenetic reprogramming of various epigenetic marks, the presence of agents that may disturb these processes, such as obesogens and other EDCs, at any of the stages may contribute to alterations in the newly determined epigenetic landscape. Since this reprogramming occurs in every generation, it is critical to determine which alterations are introduced by environmental factors and how many of these epigenomic changes can be propagated to subsequent generations in order to better understand mechanisms of transgenerational inheritance. This is currently a very controversial topic in the field (63, 65, 68, 71–73).

**Figure 1:**
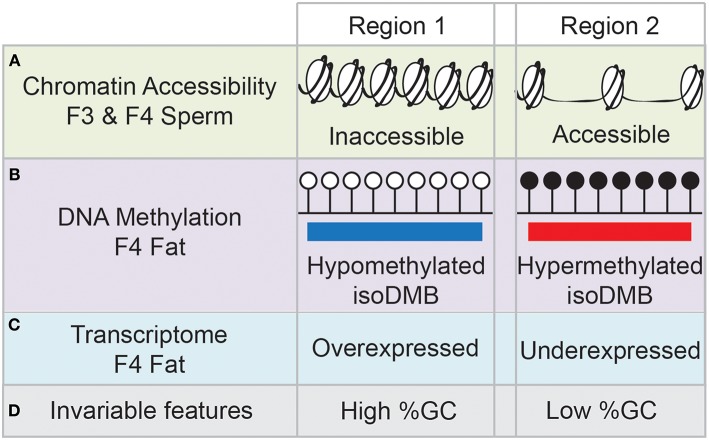
Schematic summary of the results from (29). Region 1 represents that genomic areas inaccessible in sperm samples of F3 and F4 mice **(A)** are hypomethylated in fat tissue of F4 males **(B)**, and the genes contained in those regions tend to be overexpressed **(C)**. Opposite trends are found in genomic areas represented by Region 2, with high accessibility in F3 and F4 sperm samples, and hypermethylation and underexpression in fat tissue. Genomic areas depicted by Region 1 have content of GCs, whereas genomic areas depicted by Region 2 have low GC content **(D)**.

We recently proposed a new mechanism for the transmission of epigenetic information. In our transgenerational paradigm, we exposed pregnant F0 female mice to environmentally relevant doses of the obesogen TBT throughout pregnancy (61) or throughout pregnancy and lactation (29). Following either approach, we consistently found increased adiposity in F3 and F4 males, but not females (29, 61). Interestingly, the differences in fat storage between controls and ancestrally-TBT treated animals was exacerbated when the diet of the animals was switched from a standard diet to a diet with higher fat content (29). This supports the hypothesis that ancestral exposure to TBT alters the metabolic set point of these animals multiple generations after exposure has ceased (29). Integrative methylome and transcriptome analyses of fat tissue isolated from obese F4 males did not reveal any direct associations between changes in mRNA expression levels of genes whose promoter was either hypermethylated or hypomethylated. In contrast, we found a significant number of differentially methylated regions (DMR) located in intergenic regions. We re-analyzed the data, focusing on large blocks of the genome where the alteration in DNA methylation was in the same direction (hypo- or hyper-methylated) and denoted these as **iso**directional, **d**ifferentially **m**ethylated **b**lock**s** (isoDMBs). IsoDMBs spanned large regions of the genome and we found a strong association between hypomethylated isoDMBs and over-expressed genes and between hypermethylated isoDMBs and under-expressed genes (29). Additionally, analysis of chromatin accessibility in sperm samples from F3 and F4 mice showed significant overlaps in accessibility between sperm samples in both generations (Figure 1A), suggesting that there is a conservation of the differentially accessible regions across generations. We also found an intriguing association between chromatin accessibility in sperm and isoDMBs in white adipose tissue of F4 male mice (Figure 1B). Regions that were inaccessible in sperm were hypomethylated in fat and regions that were accessible in sperm were hypermethylated in fat. We inferred that altered chromatin accessibility in the germ cells may promote or permit differential DNA methylation, epigenetic marks and gene expression in somatic tissues of the next generation (Figure 1C) (29).

We also noted that hypomethylated isoDMBs tend to be located in areas of the genome with high GC content, whereas hypermethylated isoDMBs are located in areas of the genome with low GC content (Figure 1D). In other words, rather than being located randomly distributed throughout the genome, changes in methylation were enriched in regions with specific base composition (GC content) (29). Others have shown that there is an association between base composition and higher order chromatin organization in the nucleus (74). This led us to hypothesize that *in utero* exposure to TBT causes heritable alterations in chromatin architecture that will contribute to changes in the epigenetic landscape that are reflected in the transcriptome (29). This new model provides a potential molecular mechanism that embraces previously described results showing alterations of epigenetic marks, such as DNA methylation, histone modification and expression of small non-coding RNAs in germ cells and somatic cells after ancestral exposure to EDCs such obesogens (62–64, 66, 67).

## Windows of Susceptibility

There are different windows of susceptibility to obesity after obesogen exposure. Rodents exposed to obesogens such as TBT or nonylphenol during early adulthood showed increased ectopic lipid storage in liver elevated body weight, and altered levels of metabolic hormones (52, 75). BPA or tolylfluanid exposure led to alterations in glucose metabolism and adiposity in adult mice (76, 77). Some of the metabolic alterations are only observed when the animals are exposed to high fat diets or high fat/high sugar diets (75, 77), suggesting that obesogen exposure may be increasing susceptibility to obesity in the presence of other metabolic challenges. This has important implications for the human obesity pandemic.

Exposures during *in utero* development have the potential to affect critical developmental steps that may have dramatic effects not only in the offspring, but in subsequent generations. Two different approaches have been used to study the effects of obesogens within early developmental windows: exposure of females throughout pregnancy (from fertilization to birth) (29, 37, 61) and exposure at discrete embryonic stages (E8–E14) (62–64, 67). The objective of the latter is to expose the embryos only during the time the primordial germ cells (PGCs) are traveling to the genital ridge, and during which DNA methylation is largely erased (70). However, the benefit of studying the former approach is that it allows the exposure to obesogens throughout the different phases of embryonic development, all of which may be important for obesity in later generations.

Another important factor that must be considered when assessing the effect of environmental pollutants using animal models is the concentration the animals are receiving. In order to be able to extrapolate the information obtained from animal studies to humans, it is necessary to work with concentrations of EDCs that are in the realm of what human exposure is measured or estimated to be. Typically, the chemical doses at which the endocrine system is altered are significantly lower than the concentrations that induce toxic effects in the body (44).

## Summary and Future Directions

Since the obesogen hypothesis was first proposed, studies in many laboratories have supported the existence and effects of obesogens (78). The list of *bona fide* obesogens (those shown to influence obesity, *in vivo*) continues to grow and mechanisms through which these chemicals act to promote obesity are emerging. Experimental approaches to study these mechanisms are improving. *In vitro* studies continue to provide a strong foundation to evaluate the effects of obesogens in cells, particularly as more investigators adopt stem cell models, over the limited studies possible in 3T3-L1 preadipocytes. These cell culture-based studies offer the possibility to increase the numbers of chemicals screened and may reveal new mechanisms or hypotheses about the effects of these chemicals using *in vivo* animal models. Current technology based on deep sequencing in bulk or in single cells will allow extensive whole-genome analyses that can provide information critical to understanding the epigenomic and transcriptomal state of the cells in different tissues and life stages. A major challenge in this area will be to integrate and interpret these large, multi-omic data sets to provide the most useful information.

Another major obstacle in understanding the effects of EDCs and obesogens in humans is the paucity of exposure data, particularly data from longitudinal prospective cohort studies. In addition, humans are exposed to mixtures of EDCs throughout the life course. Fortunately, experimental researchers and epidemiologists are teaming up and the advent of “exposome” studies that assess and analyze levels of exposure to obesogens together with a multitude of other chemicals, including EDCs, will enable strong mechanistic links to be made between chemical exposure and human disease (79). This will inform and guide future laboratory and epidemiological approaches that will overcome the current limitations. In turn, this will allow us to assess the costs to society of EDC and obesogen exposure more accurately. In an optimistic view, this information might influence policy makers to take appropriate steps to protect the public health.

## Author Contributions

RC-G and BB participated in the discussion of and wrote the ideas reflected in this review.

### Conflict of Interest Statement

The authors declare that the research was conducted in the absence of any commercial or financial relationships that could be construed as a potential conflict of interest.
